# Healthier diet quality and dietary patterns are associated with lower risk of mobility limitation in older men

**DOI:** 10.1007/s00394-018-1786-y

**Published:** 2018-07-23

**Authors:** Tessa J. Parsons, Efstathios Papachristou, Janice L. Atkins, Olia Papacosta, Sarah Ash, Lucy T. Lennon, Peter H. Whincup, Sheena E. Ramsay, S. Goya Wannamethee

**Affiliations:** 1 UCL Department of Primary Care and Population Health, UCL Medical School, Rowland Hill Street, London, NW3 2PF UK; 2 Psychology and Human Development, UCL Institute of Education, 25 Woburn Square, London, WC1H 0AA UK; 3 Epidemiology and Public Health Group, Medical School, University of Exeter, RILD Building, Barrack Road, Exeter, EX2 5DW UK; 4 Population Health Research Institute, St George’s University of London, Cranmer Terrace, London, SW17 0RE UK; 5 Institute of Health and Society, Newcastle University, Baddiley-Clark Building, Newcastle upon Tyne, NE2 4AX UK

**Keywords:** Diet quality, Dietary pattern, Mobility limitations, Disability, Ageing, Older adults, Men

## Abstract

**Purpose:**

To investigate associations between diet quality, dietary patterns and mobility limitation 15 years later in a population-based sample of older British men.

**Methods:**

We used longitudinal data from 1234 men from the British Regional Heart Study, mean age 66 years at baseline. Mobility limitation was defined as difficulty going up- or downstairs or walking 400 yards as a result of a long-term health problem. Dietary intake was measured using a food frequency questionnaire data from which the Healthy Diet Indicator (HDI), the Elderly Dietary Index (EDI), and three a posteriori dietary patterns were derived. The a posteriori dietary patterns were identified using principal components analysis: (1) high fat/low fibre, (2) prudent and (3) high sugar.

**Results:**

Men with greater adherence to the EDI or HDI were less likely to have mobility limitation at follow-up, top vs bottom category odds ratio for the EDI OR 0.50, 95% CI 0.34, 0.75, and for the HDI OR 0.55, 95% CI 0.35, 0.85, after adjusting for age, social class, region of residence, smoking, alcohol consumption and energy intake. Men with a higher score for the high-fat/low-fibre pattern at baseline were more likely to have mobility limitation at follow-up, top vs bottom quartile odds ratio OR 3.28 95% CI 2.05, 5.24. These associations were little changed by adjusting for BMI and physical activity.

**Conclusion:**

Our study provides evidence that healthier eating patterns could contribute to prevention or delay of mobility limitation in older British men.

**Electronic supplementary material:**

The online version of this article (10.1007/s00394-018-1786-y) contains supplementary material, which is available to authorized users.

## Introduction

Mobility limitation is one aspect of disability and occurs frequently in older adults. Disability is a broad term, defined by the World Health Organisation (WHO) as “covering impairments, activity limitations, and participation restrictions. An impairment is a problem in body function or structure; an activity limitation is a difficulty encountered by an individual in executing a task or action; while a participation restriction is a problem experienced by an individual in involvement in life situations.” As the WHO definition explains, disability is, therefore, “not just a health problem” but “a complex phenomenon, reflecting the interaction between features of a person’s body and features of the society in which he or she lives [1]”. With globally ageing populations, the burden of disability, both to individuals and healthcare systems is increasing. In 2015–2016 in the UK, 44% of adults of state pension age (≥ 63 years for women, ≥ 65 years for men) were identified as having a disability, reported as any physical or mental health condition or illness that lasts or is expected to last 12 months or more, and which limits ability to carry out day-to-day activities [2]. Mobility limitations are even more common; data from the English Longitudinal Study of Ageing suggest that 62% of adults over 60 years have at least one of a number of mobility limitations with 11% having difficulty walking 100 yards and 14% having difficulty climbing one flight of stairs [3]. Chronic conditions, for example, cardiovascular disease, diabetes, obesity, falls, hearing or visual impairments, which are more common in older adults, may contribute to disability and/or mobility limitations. A wide range of physiological changes that occur as part of the normal ageing process also have the potential to contribute to disability either directly or via chronic conditions, for example, reduction in muscle and bone strength and mass, reduced ability to control blood pressure in response to postural change, nervous system changes and deterioration in vision and hearing.

It has been suggested that disability may be delayed or at least partly prevented by being physically active and/or eating a healthy diet. Disability may be measured in a variety of ways, for example, asking individuals to report whether their health limits various physical activities, activities of daily living (e.g. washing and eating), instrumental activities of daily living (activities required to function in the community such as shopping) or social activities, or a combination of these. A number of studies have investigated the influence of diet on disability and in particular, greater adherence to the Mediterranean Diet [4–7] or higher fruit and vegetable intakes [8–12] have been reported to be related to lower risks of disability in cross-sectional and longitudinal studies. However, the number of studies, particularly in older adults, is limited, and studies vary in terms of dietary and disability measures. For example, in the Nurses Health Study in the US, a greater adherence to the Alternative Healthy Eating Index (AHEI) was associated with a lower risk of impaired physical functioning 18 years later [13]. In this study, the association was strongest for overall adherence to the AHEI diet pattern, suggesting that the overall diet pattern was more important than the top contributing foods or food groups (fruits and vegetables). In a study of Spanish older adults, one measure of adherence to the Mediterranean Diet was robustly associated with physical functioning whereas another was not [14] illustrating the complexity of defining dietary patterns. Eating patterns are often specific to time and place, and evolve over time, contributing to ongoing debate of measurement methods. Mobility limitations are most commonly operationally defined as difficulty in walking ¼ mile, or climbing a flight of stairs [15], but may also include the ability to get up from a bed or chair [16]. Walking ¼ mile (400 yards or 2–3 blocks) has especially been suggested as a useful indicator of mobility [17] since a person who can walk this distance can typically get to a shop, visit a neighbour or access public transport, whereas someone who cannot is more likely to become isolated and vulnerable. Difficulty or inability to walk ¼ mile has been shown to be associated with earlier work exit [18], increased healthcare costs, hospitalisations, new functional disability and mortality [19].

In this study, we investigated whether several dietary measures were associated with mobility limitation, as indicated by difficulty in walking 400 yards or going up- or downstairs, in older British men living in the community. We examined two a priori dietary measures, scores which assess adherence to a healthy diet or nutrient guidelines based on current evidence, and also a posteriori dietary patterns which are derived from the data, characterising the eating behaviour of a population. We used the Healthy Diet Indicator (HDI), based on WHO dietary guidelines, and the Elderly Dietary Index (EDI), based on a Mediterranean-style dietary intake as a priori measures of diet quality. The EDI was previously shown to be associated with increased risk of both all-cause mortality and cardiovascular disease mortality in this cohort, and questions have been raised as to whether diet quality scores based on food groups (such as the EDI) are more useful than those based on nutrients (such as the HDI). Adherence to a Mediterranean type diet in the UK is typically low, and we, therefore, used both measures. We also examined a posteriori dietary patterns in our data, with a prudent dietary pattern, a high-fat/low-fibre pattern and a high-sugar pattern being identified. In addition, we investigated whether any associations were mediated via BMI or physical activity, influenced by inflammation, as indicated by CRP level, or independent of the development of coronary heart disease.

## Methods

### Sample

The British Regional Heart Study is described in detail elsewhere [20]. Briefly, 7735 men were recruited from primary care practices in 24 British towns in 1978–1980 and followed prospectively. This study uses lifestyle and dietary data collected in 1998–2000 (our baseline here), when the men were aged 58–79 years and of 5516 surviving men, 4252 attended an examination and completed a lifestyle questionnaire and a food frequency questionnaire (FFQ) [21]. 837 men self-identified as having a mobility limitation (11% of 4174 providing data) and were excluded. We used follow-up data for mobility limitation in 2014 (approximately 15 years later) when 1655 of 2568 survivors completed a postal questionnaire at age 74–94 years. Of 1655 who returned a questionnaire, 1316 provided information for mobility limitations, of whom 1274 also had a HDI score, 1236 an EDI score and 1250 data for a posteriori dietary patterns. The National Research Ethics Service (NRES) Committee London provided ethical approval. Participants provided informed written consent to the investigation in accordance with the Declaration of Helsinki.

### Mobility limitation

In self-completed questionnaires at baseline and follow-up, men reported whether they had difficulty with going up- or downstairs or with walking 400 yards as a result of a long-term health problem. Men responding yes to either of these questions were defined as having a mobility limitation. At follow-up, 289 men were identified as having mobility limitations, of 1316 providing information.

### Dietary assessment

Dietary intake was measured using a self-administered FFQ, developed for use in the World Health Organisation’s Monitoring Trends and Determinants in Cardiovascular Disease Survey [22], and validated against weighed intake in British populations [23, 24]. Participants reported how often they consumed 86 food and drink items per week, selecting 1 of 9 categories: 1, 2, 3, 4, 5, 6 or 7 days/week, monthly or rarely/never. Additional information was collected on type and quantity of fruit eaten and types of fat used. The number of men with information for each of the dietary indices varies slightly due to men having missing data on some dietary questions and not others, and different variables being used to construct the scores.

### A priori diet quality scores

From the FFQ we derived two a priori pre-defined diet quality scores, the Healthy Diet Indicator (HDI) and the Elderly Dietary Index (EDI) with slight modifications, [25] (online supplement Table 1). Briefly, the HDI was originally developed using the WHO dietary guidelines for preventing chronic diseases [26] and consists of nine components, seven nutrients and two food groups (saturated fatty acids, polyunsaturated fatty acids, protein, carbohydrates, sugar, fibre, cholesterol, pulses/nuts/seeds, and fruit/vegetables), each scoring 1 if the dietary guideline was met and 0 otherwise [27]. We omitted pulses/nuts/seeds from the HDI because we did not have data for this component; therefore, the total score ranged from 0 to 8. For fruit and vegetables, we substituted frequency of consumption for weight of consumption of both fruit and vegetables consumed daily for ≥ 400 g per day and fruit and vegetables consumed less than daily for < 400 g per day because weight was unavailable in our study. We also modified cut-off points for polyunsaturated fatty acids and fibre intake for use in a British population, as suggested previously [28]. The EDI was based on the US Modified MyPyramid for Older Adults and additional recommendations [29], and consists of nine food groups (meat, fish and seafood, legumes, fruit, vegetables, cereals, bread, olive oil, and dairy). Each food group was assigned 1–4-points based on optimal consumption frequency, 1 point being least healthy and 4 points being most healthy (online supplement Table 2), which takes into consideration the U-shaped relation between certain food items and the risk of health outcomes [29]. For example, meat eaten ≥ 3 days per week scored 1 (least healthy) and meat eaten 1–2 days per week scored 4 (most healthy). The total score ranged from 9 to 36. Thus, higher scores on both the HDI and EDI indicated a healthier diet.

### A posteriori dietary patterns

We also identified a posteriori dietary patterns from the FFQ using principal component analysis, as used previously in this cohort [30]. The 86 items from the FFQ were aggregated into 34 mutually exclusive food groups and individual food items were summed to generate a total score for each of these groups. Principal component analysis was conducted using orthogonal varimax rotation on the food groups, with the food groups transformed to *z* scores to account for different scales of measurement [31]. Three a posteriori dietary patterns were identified: (1) a high-fat/low-fibre pattern, (2) a prudent dietary pattern and (3) a high-sugar pattern [30]. The ‘high-fat/low-fibre’ pattern was characterised by a high consumption of red meat, meat products, fried potato, white bread, eggs and beer (positive scoring coefficients) and a low intake of wholemeal bread (negative scoring coefficients) and explained 7.9% of the total variance. The ‘prudent’ dietary pattern was characterised by a high consumption of poultry, fish, vegetables, legumes, fruits, pasta and rice, wholemeal bread, eggs, sauces, soups and olive oil (positive scoring coefficients) and explained 7.1% of the variance. The ‘high-sugar’ pattern was characterised by a high consumption of breakfast cereals, full-fat cheese, biscuits, puddings, chocolates, sweets and sweet spreads (positive scoring coefficients) and a low consumption of beer (negative scoring coefficients) and explained 5.8% of the total variance. Higher scores indicated higher adherence to the pattern [30]. Total energy intake was estimated using a computer program which multiplied the food frequency of the food consumed by the standard portion sizes for each food, and by the nutrient composition of the food obtained from the UK food composition tables [32].

### Covariates

Social class was categorised as manual and non-manual, based on longest held occupation at study entry (1978–1980). Region of residence (1978–1980) was grouped into Scotland, North, Midlands and South of England. All other covariates were measured in 1998–2000, at the same time as dietary intake. Body mass index (BMI, kg/m^2^) was calculated from measured height and weight. Smoking, alcohol consumption and physical activity were reported by self-complete questionnaire. Current physical activity was classified into six groups on the basis of intensity and frequency of exercise (inactive; occasional; light; moderate; moderately vigorous; vigorous), a classification validated in this cohort against heart rate and forced expiratory volume in 1 s [33]. Low physical activity in this study consists of the inactive and occasional groups above. Fasting venous blood samples were analysed for CRP (mg/L) using ultrasensitive assay on an automated clinically validated analyser (e411; Roche, Burgess Hill, UK) using the manufacturer’s calibrators and controls (coefficient of variation 6.9%).

### Statistical analyses

Men defined as having a mobility limitation at baseline were excluded (*n* = 837). We calculated baseline descriptive characteristics by mobility limitation at follow-up, approximately 15 years later, and used logistic regression models to investigate associations between dietary quality or pattern at baseline and presence of mobility limitation at follow-up. The distribution of CRP was right skewed and, therefore, log transformed. Associations were considered significant where the probability was at the 5% level or below. The HDI and EDI scores were analysed as four groups of equal size as possible, for the EDI score 9–22, 23–24, 25–26, 27–36 and for the HDI score 0–1, 2, 3, 4–8. The a posteriori dietary patterns were classified as quartiles, higher quartiles indicating greater adherence to the pattern. *p* for trend was calculated by including the categories or quartiles of scores in the model as a continuous rather than categorical variable. All models were adjusted initially for age, social class, region of residence, smoking, alcohol consumption, and energy intake (Model 1) and then additionally for BMI and physical activity (Model 2). As a sensitivity analysis, we excluded men who reported ever receiving a diagnosis of angina or heart attack (coronary thrombosis or myocardial infarction) at follow-up. All analyses were conducted using Stata 14.2 (StataCorp LLC).

## Results

Men who had a mobility limitation at follow-up were older at baseline, had higher mean BMI and CRP level, were more likely to be of manual social class, be a current/recent or long-term ex-smoker and more likely to report low physical activity (Table 1). Men in the lower (less healthy) categories of the HDI or EDI had higher energy intakes, were more likely to be of manual social class, smoke, be defined as obese (BMI > 30 kg/m^2^) and for the EDI were older and more likely to be physically inactive, as has been previously reported in this cohort [25].

**Table 1:** Baseline characteristics of British men by mobility limitation at follow-up

Baseline characteristic	No mobility limitation at follow-up	Mobility limitation at follow-up	*p*	*N* ^a^
Age (years)	65.5 (4.2)	67.1 (4.8)	< 0.0001	1250
Manual social class (%)	40.6	51.5	0.001	1242
Smoker (%)	6.8	12.8	0.001	1235
Low physical activity^b^ (%)	21	28	0.01	1221
Alcohol (units per week)	11.7 (9.8)	11.2 (10.0)	0.4	1234
Energy Intake (kcal/day)	2113 (497)	2093 (518)	0.6	1244
BMI (kg/m^2^)	26.3 (3.0)	27.8 (3.6)	< 0.0001	1247
CRP (mg/l)^c^	1.20 (2.77)	1.72 (2.97)	< 0.0001	1197

Values are mean (SD) unless stated otherwise

^a^Men with data on a posteriori dietary patterns at baseline and mobility limitation at follow-up

^b^Inactive or occasionally active based on intensity and frequency of exercise reported by questionnaire

^c^Geometric means given due to skewed data

Men who were lost to follow-up were less healthy than those with follow-up data; of men who did not have mobility limitation data at follow-up, 25% had a mobility limitation at baseline and 21% were in the top category for the EDI score, compared with 12% with a mobility limitation at baseline and 30% in the top category for the EDI score for men with mobility limitation data at follow-up.

Men with a greater adherence to the EDI at baseline were less likely to have a mobility limitation at follow-up, top vs bottom category OR 0.50, 95% CI 0.34, 0.75, after adjusting for age, social class, region of residence, smoking, alcohol consumption and energy intake (Table 2, model 1). Similarly, men with a greater adherence to the HDI at baseline were less likely to have a mobility limitation at follow-up, top vs bottom category OR 0.55, 95% CI 0.35, 0.85, after adjusting for the same covariates. Men with a higher score for the high-fat/low-fibre pattern at baseline were more likely to have a mobility limitation at follow-up, top vs bottom quartile OR 3.28, 95% CI 2.05, 5.24 (Table 2, model 1). The magnitude of these associations was slightly reduced on adjustment for BMI and physical activity but remained significant (Table 2, model 2).

**Table 2:** Prospective associations between dietary quality or patterns at baseline and mobility limitation at follow-up, odds ratios and 95% confidence intervals

	Model 1		Model 1		Model 2		Model 2 excluding angina and heart attack^a^		Model 3	
	*N*	Cases (*n*)	OR	(95% CI)	OR	(95% CI)	OR	(95% CI)	OR	(95% CI)
EDI categories										
Score 9–22	260	82	1.00		1.00		1.00		1.00	
Score 23–24	315	72	0.70	(0.48, 1.03)	0.67	(0.45, 1.00)	0.73	(0.46, 1.16)	0.68	(0.45, 1.02)
Score 25–26	271	53	**0.62**	**(0.41, 0.94)**	**0.61**	**(0.40, 0.94)**	**0.58**	**(0.34, 0.98)**	0.66	(0.43, 1.02)
Score 27–28	388	62	**0.50**	**(0.34, 0.75)**	**0.53**	**(0.35, 0.80)**	**0.47**	**(0.28, 0.78)**	**0.53**	**(0.35, 0.82)**
			*n* = 1234	*p* = 0.001	*n =* 1207	*p* = 0.003	*n* = 943	*p* = 0.003	*n* = 1158	*p* = 0.006
HDI categories										
Score 0–1	164	47	1.00		1.00		1.00		1.00	
Score 2	242	66	0.92	(0.58, 1.45)	0.97	(0.61, 1.55)	0.91	(0.53, 1.55)	0.85	(0.53, 1.38)
Score 3	360	68	**0.56**	**(0.36, 0.88)**	**0.60**	**(0.38, 0.95)**	**0.53**	**(0.31, 0.90)**	**0.56**	**(0.35, 0.89)**
Score 4–8	436	80	**0.55**	**(0.35, 0.85)**	**0.61**	**(0.39, 0.96)**	**0.41**	**(0.24, 0.70)**	**0.49**	**(0.31, 0.78)**
			*n* = 1202	*p* = 0.001	*n* = 1176	*p* = 0.006	*n* = 917	*p* < 0.0001	*n* = 1127	*p* < 0.0001
High-fat/low-fibre pattern quartiles										
1	382	60	1.00		1.00		1.00		1.00	
2	316	57	1.17	(0.78, 1.76)	1.07	(0.70, 1.63)	1.15	(0.68, 1.94)	1.08	(0.70, 1.66)
3	274	68	**1.77**	**(1.17, 2.68)**	1.50	(0.98, 2.30)	1.47	(0.86, 2.51)	1.53	(0.99, 2.37)
4	237	82	**3.28**	**(2.05, 5.24)**	**2.76**	**(1.70, 4.48)**	**3.22**	**(1.77, 5.86)**	**2.74**	**(1.65, 4.54)**
			*n* = 1209	*p* < 0.0001	*n* = 1183	*p* < 0.0001	*n* = 920	*p* ≤ 0.0001	*n* = 1134	*p* < 0.0001
Prudent dietary pattern quartiles										
1	256	65	1.00		1.00		1.00		1.00	
2	297	78	1.14	(0.77, 1.70)	1.21	(0.81, 1.83)	1.48	(0.91, 2.42)	1.30	(0.85, 1.97)
3	323	61	0.83	(0.54, 1.26)	0.86	(0.56, 1.33)	0.92	(0.54, 1.55)	0.90	(0.57, 1.41)
4	333	63	0.86	(0.55, 1.33)	0.90	(0.57, 1.41)	0.98	(0.55, 1.72)	0.94	(0.59, 1.50)
			*n* = 1209	*p* = 0.2	*n* = 1183	*p =* 0.3	*n* = 920	*p* = 0.5	*n* = 1134	*p* = 0.4
High-sugar pattern quartiles										
1	275	72	1.00		1.00		1.00		1.00	
2	316	67	0.75	(0.50, 1.12)	0.78	(0.51, 1.17)	1.03	(0.62, 1.71)	0.75	(0.49, 1.14)
3	300	63	0.70	(0.46, 1.07)	0.76	(0.49, 1.18)	0.92	(0.54, 1.58)	0.80	(0.51, 1.25)
4	318	65	0.68	(0.42, 1.12)	0.71	(0.43, 1.18)	0.81	(0.43, 1.53)	0.71	(0.42, 1.20)
			*n* = 1209	*p* = 0.1	*n* = 1183	*p* = 0.2	*n* = 920	*p* = 0.5	*n* = 1134	*p* = 0.3

*HDI* Healthy Diet Indicator, *EDI* Elderly Dietary Index

Values are odds ratios (95% confidence intervals) for categories/quartiles 2, 3 and 4 compared to category/quartile 1

Bold type indicates significance at the 5% level

*p* value for trend

Model 1: adjusted for age, social class, region of residence, smoking, alcohol consumption, energy intake

Model 2: adjusted as for model 1 plus BMI, physical activity

Model 3: adjusted as for model 2 plus C-reactive protein (CRP)

^a^Coronary thrombosis or myocardial infarction

Both BMI and physical activity contributed to the attenuation, with BMI having a greater effect (data not shown). Additional adjustment for CRP did not change findings (Table 2, model 3). Excluding men who at follow-up reported ever having had angina or heart attack strengthened associations (Table 2, model 2 excluding angina and heart attack). We found no association between the prudent diet pattern and the high-sugar diet pattern and mobility limitation.

## Discussion

Our study showed that for both pre-defined dietary quality scores, greater scores (healthier diets) were associated with a lower risk of mobility limitation in terms of difficulty walking 400 yards or going up- or downstairs 15 years later. Additional adjustment for BMI and physical activity did not change these findings. Of the a posteriori patterns, greater adherence to the high-fat/low-fibre pattern was associated with a higher risk of mobility limitation, whereas no associations were seen for the prudent dietary pattern or high-sugar pattern. Few existing studies have focused on mobility limitations, despite measures such as the ability to walk 400 yards being strong predictors of future health and function [34] and few studies have investigated multiple measures of diet quality in relation to disability. The EDI is related to the Mediterranean Diet Score in terms of food groups emphasised. It was based on the Modified MyPyramid for Older Adults [29], which in turn was based on US Dietary Guidelines and recommendations made by other health organisations encouraging diets high in whole grains and cereals, vegetables, fruits, low-fat dairy products, legumes, fish, and lean meats. The EDI additionally incorporates olive oil and also alcohol (alcohol was omitted in our study and others). The HDI is based more on nutrient intake than food groups (in our study saturated fatty acids, polyunsaturated fatty acids, protein, carbohydrates, sugar, fibre, cholesterol, and fruit/vegetables), and uses a binary cut-off for each component which is cruder than the 4-point ranges used in the EDI. It has been argued that dietary indices based on food groups may be more useful than those based on nutrient guidelines; they may be easier to measure and incorporate less error given that the conversion of FFQ data into nutrient intake involves assumptions about portion sizes and is dependent on a current and comprehensive food database. However, in our population of older men, both the EDI and HDI showed associations with mobility limitation. Some or all of the nutrients and food groups incorporated into the EDI and HDI may be related to disability and/or mobility limitation.

There is a wide range of mechanisms by which various food groups and nutrients might affect aspects of disability, and these might operate directly or indirectly via other chronic diseases. Ageing is associated with higher levels of oxidants and free radicals which in excess lead to oxidative stress which is detrimental to cell function [35] and can promote inflammation [35, 36]. Berries, fruits, nuts, chocolate, vegetables and also some legumes and wholegrains are good dietary sources of antioxidants [37] and both higher intakes and plasma levels of antioxidants have been found to be positively associated with various measures of physical performance and strength [38]. Fruit and vegetables have been demonstrated to influence antioxidant pathways, the immune system and cholesterol and steroid hormone concentrations and metabolism [39]. Polyunsaturated and monounsaturated fatty acids have cholesterol-lowering effects, [40] and anti-inflammatory effects have been found for the phenolic compounds in olive oil, omega-3 fatty acids in oily fish [41] and tocopherols in fruits, vegetables and nuts [42]. The fibre from increased wholegrain and fruit and vegetable intake improves glucose and lipid metabolism and insulin sensitivity [43].

We also found a greater adherence to a high-fat/low-fibre dietary pattern (i.e. a less healthy diet pattern) was associated with an increased risk of mobility limitation, which is in agreement with the associations with the dietary quality indices. The prudent diet pattern and high-sugar pattern were not associated with mobility limitation, possibly because these patterns are not capturing the most relevant dietary constituents. Additionally, adjusting regression models for BMI and physical activity led to slight attenuation of the associations between the EDI, HDI or high-fat/low-fibre pattern and mobility limitation (odds ratio changed by 6, 11 and 16%, respectively), with BMI contributing more to the attenuation than physical activity. This suggests that part of the association with either the HDI or high-fat/low-fibre dietary pattern is due to BMI and physical activity, yet the dietary pattern remains important of itself. Adjusting for CRP level did not alter findings, suggesting the dietary associations are not mediated via inflammation. Previous work on this cohort shows over a slightly shorter time period that men eating more healthily on the EDI had significantly lower risks of coronary heart disease events (fatal and non-fatal) [25], which are in turn related to mobility limitations. However, excluding men who reported angina or heart attack suggested the diet–mobility limitation associations were not due to coronary heart disease.

## Strengths and limitations

Our study has several major strengths; the study sample is large and population based, and data were collected prospectively over a period of 15 years. Less healthy men were more likely to be lost to follow-up, but given that non-responders at follow-up were more likely to have a mobility limitation and eat less healthily at baseline, the associations we report are likely to be conservative. We also have information on a wide range of potential confounders. To reduce bias by reverse causation we excluded men with mobility limitation at baseline. We repeated analysis after excluding men with angina or heart failure at follow-up, to see if associations were driven by these conditions, which are known to be strongly associated with mobility limitation [44]. We used a FFQ that has been previously validated against weighed food intakes in British populations and widely used [23, 24]. Whilst FFQs may be more error prone than weighed food intakes or 24-h recalls they are less intensive for study participants, particularly older adults, and allow longer term dietary habits to be captured. The HDI and EDI are well-known diet quality scores that have been shown to be inversely associated with mortality and/or cardiovascular disease in older European cohorts. Using a priori diet quality scores allowed us to investigate whether adherence to dietary guidelines or consumption of a Mediterranean type diet is related to mobility limitations. However, whilst these scores assess adherence to healthy a diet according to current evidence, they may not reflect existing food habits in the population. The advantage of a posteriori dietary patterns is that they are derived from the data rather than based on previous assumptions, but subjective decisions such as in grouping of dietary variables and identifying components to retain are involved. Although the three dietary patterns explained a seemingly low proportion of the total variance (21%), this is not unusual; a similar study in the UK reported 10% [45] of the variance explained and a study in Hong Kong 17% [46]. We acknowledge that we have a measure of dietary intake only at follow-up, and changes in dietary habits may have occurred over time that we have not captured. We used self-reported measures for mobility limitations which may be subject to recall bias, cognitive ability and in the case of walking 400 yards, misgauging of distance. However, these are less of a burden for study participants than performance tests, have higher response rates and have been shown to predict mobility disability [15]. Our study population of older men is almost exclusively White European and, therefore, findings cannot be generalised to women, other ethnic groups or younger age groups, in whom dietary patterns may differ.

In summary, we found that greater adherence to the EDI or HDI at age 58–79 years was associated with a lower odds of mobility limitation 15 years later at age 74–94 years. In line with these findings, a greater adherence to the a posterior high-fat/low-fibre pattern was associated with a higher odds of subsequent mobility limitation. These associations were independent of socioeconomic and lifestyle factors including BMI and physical activity. Our study suggests that even in older age groups healthy eating patterns are potentially important in terms of contributing to the delay or prevention of mobility limitation.

## Electronic supplementary material

Below is the link to the electronic supplementary material.

Supplementary material 1 (DOCX 18 KB)
