# Supplementary material for: Healthier diet quality and dietary patterns are associated with lower risk of mobility limitation in older men
Source: Eur J Nutr. 2018 Jul 23;58(6):2335–43. doi: 10.1007/s00394-018-1786-y (PMC6689276; doi:10.1007/s00394-018-1786-y)
Supplement: Supplementary file 1 — Supplementary material 1 (DOCX 18 KB) [file 394_2018_1786_MOESM1_ESM.docx]

**Online Supplement Table 1. Healthy Diet Indicator components and scoring criteria**

| Healthy Diet Indicator Scoring | | |
| --- | --- | --- |
| Component | Score = 0 | Score = 1 |
| Saturated fatty acids (% energy) | >10 | 0-10 |
| Polyunsaturated fatty acids (% energy) | <6 and >10 | 6-10 |
| Protein (% energy) | <10 and >15 | 10-15 |
| Total carbohydrates (% energy) | <50 and >70 | 50-70 |
| Sugar (% energy) | >10 | 0-10 |
| Dietary Fibre (g/day) | <18 and >32 | 18-32 |
| Cholesterol (mg/d) | >300 | 0-300 |
| Fruits and Vegetables1 | Less frequent than daily consumption of both | Daily consumption of both |

**Online Supplement Table 2. Elderly Diet Index components and scoring criteria**

| **Elderly Dietary Index Scoring** | | | | |
| --- | --- | --- | --- | --- |
| Component | Score = 1 | Score = 2 | Score = 3 | Score = 4 |
| Meat | ≥3 days/week | Never/rarely | <1 day/week | 1-2 days/week |
| Fish/Seafood | Never/rarely | <1 day/week | ≥3 days/week | 1-2 days/week |
| Legumes | Never/rarely | <1 day/week | ≥3 days/week | 1-2 days/week |
| Fruit | <1 day/week | 1-2 days/week | 3-6 days/week | Daily |
| Vegetables | <1 day/week | 1-2 days/week | 3-6 days/week | Daily |
| Cereals | <1 day/week | 1-2 days/week | 3-6 days/week | Daily |
| Bread | None | White | White and whole grain | Whole grain |
| Olive oil^1^ | Never/Rarely | Tertile 1 of intake | Tertile 2 of intake | Tertile 3 of intake |
| Dairy | Full-fat milk and full-fat cheese | Semi-skimmed milk and full-fat cheese / full-fat milk and low-fat cheese | Skimmed milk and full-fat cheese | Skimmed/Semi-skimmed milk and low-fat cheese |

^1^The frequency of olive oil consumption was not available so this component was modified from the original score used (1 = <1 day/week; 2 = 1-2 days/week; 3 = 3-6 days/week; 4 = daily) to the quantity of weekly consumption (never/rarely consumed and tertiles of weekly consumption)
